# Epilepsy concordance in monozygotic twins: the role of common genetic variants

**DOI:** 10.1093/brain/awaf362

**Published:** 2025-09-25

**Authors:** Yew Li Dang, Karen L Oliver, Kate Esnault, Melanie Bahlo, Piero Perucca, Samuel F Berkovic

**Affiliations:** Department of Medicine (Austin Health), Epilepsy Research Centre, The University of Melbourne, Melbourne, Victoria 3084, Australia; Department of Neurology, Austin Health, Bladin-Berkovic Comprehensive Epilepsy Program, Melbourne, Victoria 3084, Australia; Department of Medicine (Austin Health), Epilepsy Research Centre, The University of Melbourne, Melbourne, Victoria 3084, Australia; Genetics and Gene Regulation Division, the Walter and Eliza Hall Institute of Medical Research, Parkville, Victoria 3050, Australia; Department of Medicine (Austin Health), Epilepsy Research Centre, The University of Melbourne, Melbourne, Victoria 3084, Australia; Genetics and Gene Regulation Division, the Walter and Eliza Hall Institute of Medical Research, Parkville, Victoria 3050, Australia; Department of Medical Biology, University of Melbourne, Carlton, Victoria 3010, Australia; Department of Medicine (Austin Health), Epilepsy Research Centre, The University of Melbourne, Melbourne, Victoria 3084, Australia; Department of Neurology, Austin Health, Bladin-Berkovic Comprehensive Epilepsy Program, Melbourne, Victoria 3084, Australia; Department of Neuroscience, School of Translational Medicine, Monash University, Melbourne, Victoria 3004, Australia; Department of Neurology, Alfred Health, Melbourne, Victoria 3004, Australia; Department of Neurology, The Royal Melbourne Hospital, Melbourne, Victoria 3050, Australia; Department of Medicine (Austin Health), Epilepsy Research Centre, The University of Melbourne, Melbourne, Victoria 3084, Australia; Department of Neurology, Austin Health, Bladin-Berkovic Comprehensive Epilepsy Program, Melbourne, Victoria 3084, Australia

**Keywords:** twin study, epilepsy, polygenic risk score, concordance, common variants

## Abstract

Factors underlying discordance for epilepsy in monozygotic twins, in the absence of obvious acquired insults, are incompletely understood. Whilst subtle lesions and postzygotic mutations are sometimes observed, the contribution of common genetic variants remains unexplored. We investigated the role of these variants, measured by polygenic risk scores, in epilepsy concordance. We hypothesized that higher epilepsy polygenic risk scores in concordant monozygotic twins, compared to discordant monozygotic twins and controls, reflect increased epilepsy risk, raising the likelihood of both twins being affected.

We calculated epilepsy polygenic risk scores for 102 monozygotic twin pairs (49 concordant, 53 discordant) and 14 632 controls using 2023 epilepsy genome-wide association study summary statistics. Logistic regression, adjusted for sex and principal ancestry components, showed that concordant pairs had significantly higher epilepsy polygenic risk scores than discordant pairs (mean 0.71 versus 0.18; *P*_adj_ = 0.03) and controls (mean 0.71 versus 0; *P*_adj_ = 0.001). In contrast, epilepsy polygenic risk scores in discordant pairs did not differ from controls (mean 0.18 versus 0; *P*_adj_ = 0.38).

Our findings suggest that concordance for epilepsy in monozygotic twins is partly driven by common genetic variant burden, underscoring the potential utility of epilepsy polygenic risk scores as predictive markers for epilepsy risk in the general population.

## Teaser

Dang *et al*. analysed epilepsy polygenic risk scores (PRSs) in monozygotic twins and found that twins concordant for epilepsy had higher PRSs than discordant twins and controls. These findings highlight the contribution of common genetic variants to epilepsy concordance and support the potential of PRSs as markers of disease risk.

## Introduction

Twin studies have been instrumental in elucidating the heritability of epilepsy. They have served as a powerful heuristic in epilepsy research, providing early evidence for the genetic contributions to epilepsy well before genetic discoveries.^1,2^ Concordance for epilepsy in monozygotic (MZ) twins is taken as evidence for a germline genetic contribution to epilepsy.

A notable finding from early and contemporary twin studies was the observation of MZ twins discordant for epilepsy, in the absence of acquired lesions or subtle MRI findings, and even in epilepsy syndromes where other evidence pointed strongly to genetic underpinnings, such as the idiopathic generalized epilepsies.^1,3,4^ Understanding disease discordance in MZ twins has general applicability to addressing problems of penetrance and variable expressivity in the general (non-twin) population. Postzygotic mutations,^5^ present only in the affected twins, account for discordance in epilepsy in some pairs. However, the contribution of common genetic variants to the concordance for epilepsy in MZ twins remains unexplored.

Common genetic variants in epilepsy can be aggregated into a polygenic risk score (PRS), providing a meaningful measure of the cumulative genetic risk for epilepsy. Epilepsy PRSs are enriched in individuals with epilepsy compared to population controls, indicating an increased genetic liability for epilepsy. This enrichment is observed in both sporadic and familial cases,^6-8^ and extends to individuals with known or presumed rare pathogenic variants.^9,10^ Additionally, Heyne *et al*.^11^ recently demonstrated the predictive value of genetic generalized epilepsy (GGE) PRSs. They found that for every standard deviation increase in GGE PRSs, individuals are 1.73 times more likely to develop GGE following an unspecified seizure. However, the effect size of epilepsy PRSs is relatively modest compared to the PRSs for other common conditions, such as coronary artery disease.^6,12^

The aim of this study was to analyse the association between epilepsy PRSs and the concordance for epilepsy in MZ twin pairs without obvious acquired factors or pathogenic rare variants. We compared epilepsy PRSs in epilepsy concordant MZ twin pairs (where both twins in each pair have epilepsy) to epilepsy discordant MZ twin pairs (where only one twin is affected). We hypothesized that concordant MZ pairs would have a higher epilepsy PRS, reflecting greater genetic liability for epilepsy, thus increasing the likelihood of both twins being affected. In contrast, discordant MZ twin pairs would have a lower epilepsy PRS, closer to that of the general population, increasing the risk of only one twin being affected.

## Materials and methods

### Ascertainment

This study included 169 MZ twin pairs from the Epilepsy Research Centre twin database (University of Melbourne, Australia), where at least one twin in each pair was diagnosed with epilepsy and had available DNA samples. The twins were ascertained from community-based twin registers and by referral since 1988.^3^ Twins with significant acquired insults, e.g. from perinatal or traumatic brain injury, a structural/lesional aetiology, or postzygotic mutations accounting for the discordance in epilepsy, were excluded. Twins with pathogenic rare germline variants with large effect, considered major germline contributors to epilepsy, were also excluded. Control data were obtained (n = 14 632) from the QSkin Sun and Health Study, Australia.^13^

This study was approved by the Human Research Ethics Committee of Austin Health (H2007/02961) and the Walter and Eliza Hall Institute Ethics Committee (G20/01, 24/36). Written informed consent was obtained from all twins and their guardians in the case of minors. The QSkin Sun and Health Study was approved by QIMR Berghofer Human Research Ethics Committee (P1309, P2034), including amendments to share data with this study.

### Clinical classifications

Epilepsy diagnoses and classifications were made according to the 2017 International League Against Epilepsy (ILAE) criteria,^14^ based on comprehensive clinical evaluation and consensus review of clinical data. Twins with epilepsy were categorized into three main types: Generalized Epilepsy (GE), Focal Epilepsy (FE), and Other Epilepsy, which includes individuals with mixed or unclassified epilepsy.

Twins with generalized epileptic syndromes were categorized as GE. This category included twins with idiopathic generalized epilepsy, and other generalized epilepsy syndromes, such as epilepsy with myoclonic-atonic seizures, and febrile seizures plus. Twins with focal epilepsies were classified as having FE. The Other Epilepsy category comprised twins with both focal and generalized seizures (mixed epilepsy) or recurrent seizures of unknown origin (unclassified epilepsy).

A family history of epilepsy was considered positive if epilepsy was present in first- or second-degree relatives of the proband.

### Genetic analysis

Zygosity was determined using a validated zygosity questionnaire with >95% accuracy,^15^ and subsequently confirmed via genotyping using 10 highly polymorphic short tandem repeat markers with a 0.00016 average probability that a dizygotic pair would be identical at all tested loci.^15^

Within the constraints of single-nucleotide polymorphism (SNP) genotyping error and quality control measures, MZ twins are considered to have identical SNPs.^16^ Therefore, SNP genotyping was performed on only one twin in each MZ pair. SNP genotyping was performed in the affected twins for all but three cases, where poor DNA quality in the affected twin necessitated using DNA from the unaffected twin. SNP genotyping for controls and MZ cases was performed on blood or saliva-derived DNA using the Illumina Global Screening Array (GSA) versions 1 and 3, respectively.

### Data processing

Case and control data were merged using PLINK V1.9.^17^ We excluded SNPs with high ‘missingness’ rates (>2%) or low minor allele frequency (MAF < 0.5%). We also excluded samples with more than 2% missing genotypes among all SNPs, high (>0.2) or low (<-0.2) rates of heterozygosity, and those with sex mismatch between genotyped and reported sex. Furthermore, a technical duplicate was genotyped in both the case and control cohorts, allowing us to identify and exclude SNPs that were differentially called by the two versions of the Illumina GSA platform.

Principal component analysis for ancestry was performed on our final case-control dataset using Genome-wide Complex Trait Analysis software^18^ with data from the 1000 Genomes Project. Samples that did not cluster with the 1000 Genomes European super-population were excluded. The principal components (PCs) generated were included as covariates in subsequent PRS statistical analyses.

SNP imputation was then performed using the TOPMed r3 (GRCh38) reference panel^19^ with MiniMac^20^ as implemented on the TOPMed Imputation Server.^21^ We selected high-quality imputed and genotyped SNPs (imputation quality scores R2 > 0.9).

### PRS analysis

We calculated PRSs with the PRS-CS-auto method,^22^ using summary statistics from the 2023 ILAE genome-wide association study (GWAS) to determine the SNP selection and effect sizes.^23^ Twenty-seven samples that contributed to the source GWAS were excluded from the analysis to prevent potential inflation of the calculated PRSs due to sample overlap.^24^

We considered the all-epilepsy PRS model the most relevant, as our main analysis is focused on twins with variable epilepsy types.

Using PRS-CS-auto, we also calculated the inflammatory bowel disease (IBD) PRS for twin pairs (cases) and controls as a negative control experiment, using summary statistics from an independent GWAS study.^25^

All PRS values were standardized to a normal distribution with a mean of zero and a standard deviation of one using the control cohort as reference.

### Statistical analysis

Differences in cohort characteristics, such as epilepsy types, were assessed using a chi-squared test. We then fitted a logistic regression model using R version 2024.12.1 + 563, where we treated epilepsy concordance as a categorical (binary) outcome variable, PRS as the exposure variable, and sex and the first five ancestry PCs as fixed-effects covariates. We chose the first five PCs as they explained 96% of the variance (Supplementary Fig. 1). Mean epilepsy PRSs and the standard deviations were calculated for concordant MZ twins, discordant MZ twins, and controls. These scores were compared to determine if there were significant differences among the groups (concordant, discordant, and control). We corrected for multiple comparisons using Benjamini-Hochberg correction, with an adjusted *P*-value of <0.05 considered statistically significant.

Sensitivity analyses were performed using the GGE- and FE-PRS models from the 2023 ILAE GWAS on the entire cohort,^23^ followed by stratification based on GE and FE types. As these analyses were exploratory and involved small sample sizes within each group when stratified by epilepsy type, *P*-values were not adjusted for multiple comparisons.

## Results

### Cohort

**Figure 1:**
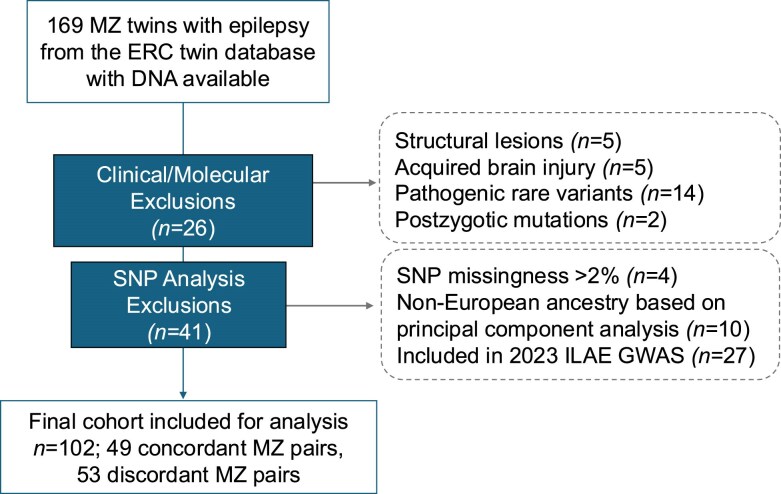
**Process of review and exclusion of twin pairs based on sample and clinical factors.** ERC = Epilepsy Research Centre; GWAS = genome-wide association study; ILAE = International League Against Epilepsy; MZ = monozygotic; SNP = single-nucleotide polymorphism.

The final cohort included for analysis comprised 102 (49 concordant, 53 discordant) MZ twin pairs. Figure 1 presents a flowchart illustrating the number of twins excluded and the reason for exclusion from the final cohort. Of these, 67 (66%) of 102 twins underwent rare variant testing. The cohort consisted of more females (62 females versus 40 males); however, there was no significant difference in the proportion of females to males among the concordant and discordant MZ pairs (Table 1).

**Table 1:** Cohort characteristics

	Total number of twin pairs	Number of concordant twin pairs (%)	Number of discordant twin pairs (%)
Monozygotic twins with epilepsy	102	49 (48%)	53 (52%)
Sex			
Male	40	22 (55%)	18 (45%)
Female	62	27 (44%)	35 (56%)
Epilepsy type			
Generalized epilepsy	42	27 (64%)	15 (36%)
Focal epilepsy	43	17 (40%)	26 (60%)
Other epilepsy	17	5 (29%)	12 (71%)
Family History of Epilepsy (1st and 2nd degree)	34	17 (50%)	17 (50%)

There was a similar number of MZ twin pairs with GE (*n* = 42) and FE (*n* = 43). Among these, 64% of GE twin pairs were concordant for epilepsy, compared to 40% of FE pairs. Given the significant difference (*P* = 0.04) in the proportion of concordant pairs between the GE and FE groups, we performed a sensitivity analysis using all epilepsy-, GGE- and FE-PRS models, incorporating epilepsy type as a covariate in the logistic regression analysis (Supplementary Table 1).

Additionally, there was an equal number of concordant and discordant pairs with a family history of epilepsy. The control cohort comprised 14 632 Australian individuals, of whom 6620 (45%) were males.

### Epilepsy PRSs

**Figure 2:**
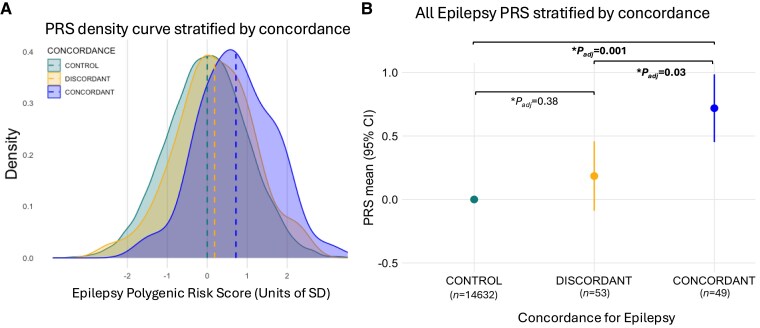
**PRS density curve and all epilepsy PRS stratified by concordance.** (**A**) Normalized distributions of epilepsy polygenic risk scores (PRSs) in concordant twin pairs (in blue) compared to discordant twin pairs (in yellow) and population controls (in green). (**B**) Mean epilepsy PRS values with 95% confidence intervals for the twin cohort, stratified by concordance for epilepsy. The PRSs were standardized to a normal distribution with a mean of 0 and a standard deviation (SD) of 1 in the control group. In the logistic regression model, the outcome variable was the concordance for epilepsy, with the epilepsy PRS as the exposure variable. The model also included sex and the first five principal components of ancestry as covariates. An adjusted *P*-value of <0.05 was considered statistically significant. CI = confidence interval.

Using the all-epilepsy PRS model, we analysed the entire cohort regardless of epilepsy type. We found that MZ twin pairs concordant for epilepsy exhibited significantly higher epilepsy PRSs compared to discordant MZ twins (mean PRS 0.71 versus mean PRS 0.18; *P*_adj_ = 0.03) (Fig. 2). For every one-standard deviation increase in epilepsy PRS, MZ twin pairs were 13% more likely to be concordant for epilepsy (OR = 1.13, 95% CI: 1.02–1.25; *P*_adj_ = 0.03 Wald test). When compared to population controls, the mean epilepsy PRS for the concordant MZ pairs was significantly higher (mean PRS 0.71 versus mean PRS 0; *P*_adj_ = 0.001). In contrast, the mean epilepsy PRSs for the discordant MZ pairs (mean PRS 0.18; *P*_adj_ = 0.38) were lower and did not differ from population controls.

In our primary analysis, where we examined the entire cohort regardless of epilepsy type, we considered the all-epilepsy PRS model the most relevant due to its inclusion of all epilepsy types. In the sensitivity analysis, the GGE-PRS model showed similar trends—higher PRS in concordant versus discordant twins (Supplementary Fig. 2)—and effect sizes comparable to the all-epilepsy PRS model (weight [beta] for GGE-PRS 0.14, weight for all epilepsy PRS 0.12) (Supplementary Table 2), reflecting overlapping SNPs between these models. While the FE-PRS model also demonstrated similar trends, the effect sizes were smaller (weight for FE-PRS 0.05).

Using the all-epilepsy PRS model, the positive trend of higher PRSs observed in concordant MZ pairs compared to discordant MZ pairs was consistent across all epilepsy types (Supplementary Fig. 3). When stratifying twin pairs by epilepsy type and analysing with the GGE-PRS (Supplementary Fig. 4) and FE-PRS models (Supplementary Fig. 5), no statistically significant differences in mean GGE-PRS or FE-PRS were observed between concordant and discordant pairs within each epilepsy type.

In the negative control experiment, using IBD PRS, no significant differences in IBD PRS scores were identified between concordant MZ twins, discordant MZ twins, or controls (Supplementary Fig. 6).

## Discussion

Our data reveals differences in genetic liability for epilepsy, as measured by epilepsy PRSs, between concordant and discordant MZ twin pairs. MZ twin pairs with a higher epilepsy PRS are more likely to have an affected co-twin, underscoring the influence of common genetic variants on epilepsy risk. In contrast, MZ twin pairs with a lower epilepsy PRS, which do not significantly differ from those of the population control, are more likely to be discordant for epilepsy.

Our findings are best conceptualized within the framework of the liability threshold model.^26^ This model posits that individuals whose liabilities—derived from a combination of genetic and other factors—exceed a certain threshold will develop the condition. Significant factors, such as pathogenic rare variants of large effect or significant acquired insults, such as those from perinatal or traumatic brain injuries, can push individuals beyond this threshold.

**Figure 3:**
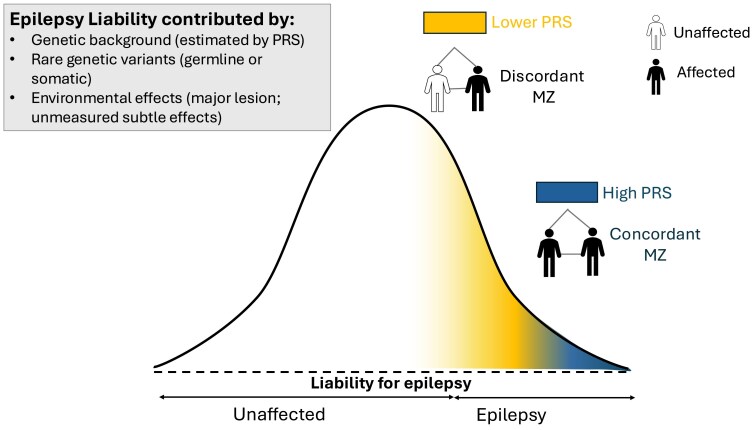
**Liability threshold model curve for epilepsy.** Individuals with a liability exceeding the threshold for epilepsy would develop epilepsy.^26^ This liability can be influenced by genetic factors (e.g. rare pathogenic variants)^27^ and non-genetic factors such as acquired brain injuries.^1,28^ Concordant monozygotic (MZ) twin pairs have a higher epilepsy polygenic risk score (PRS) that portends an increased liability for epilepsy, exceeding that of the threshold for epilepsy, resulting in both twins in each pair being affected. In contrast, discordant MZ pairs have a lower epilepsy PRS, denoting a lower liability for epilepsy, which sits just at or below the threshold for epilepsy, with only one twin affected in the presence of additional unmeasured minor acquired factors, non-shared environmental factors, or postzygotic mutation. CI = confidence interval.

Our results suggest that common genetic variants contribute to the liability threshold for epilepsy (Fig. 3). Concordant MZ pairs exhibit liabilities that exceed the threshold for epilepsy, primarily driven by common genetic variants, resulting in both twins being affected. In contrast, discordant MZ twin pairs have liabilities that are at or below this threshold. In discordant pairs, only the affected twin exceeds the threshold for epilepsy, likely due to the additional influence of postzygotic mutations and unmeasured minor acquired or non-shared environmental factors.

Common genetic variants for epilepsy contribute to the concordance for epilepsy in MZ twins. This is consistent with existing evidence that genetic background modifies epilepsy risk. Our study demonstrates differences in common genetic variants between concordant and discordant MZ twins—a finding that is novel in epilepsy but has been observed in other medical conditions. For instance, Johnston *et al*.^29^ found that MZ twins who were concordant for Type 1 Diabetes Mellitus (T1DM) are more likely to carry both HLA-DR3 and -DR4 antigens compared to discordant MZ pairs. This finding suggests that compound heterozygotes for HLA-DR3 and -DR4 confer a greater risk for T1DM, with these alleles now recognized as relevant risk factors for the development of T1DM.^29^ More recently, data from the Swedish Twin Registry demonstrated that MZ twin pairs concordant for psychosis had a higher PRS for the condition compared to discordant MZ pairs.^30^ This supports the role of PRS for psychosis as a marker of genetic susceptibility for psychosis and its utility in predicting concordance for psychosis among MZ twins.

### Limitations

Our study has limitations. First, the small sample size limits our ability to detect significant differences in epilepsy PRSs between concordant and discordant MZ pairs when stratified by epilepsy type. In the sensitivity analysis, when twin pairs were stratified by epilepsy type, the previously observed significant difference in the analysis of the entire cohort was no longer present using the all-epilepsy, GGE-PRS and FE-PRS models. Additionally, despite a nominal difference in mean epilepsy PRSs between concordant and discordant pairs within the Other Epilepsy group in the all epilepsy- and FE-PRS models, 95% confidence intervals were wide due to the small number of pairs, and no between-group differences reached statistical significance.

Although prior research suggests that GGE-PRS and FE-PRS are enriched within their respective epilepsy types,^6^ we did not observe any significant differences in effect sizes between GE and FE twins using both the GGE-PRS and FE-PRS models. This may be due to the limited number of twin pairs within each epilepsy type in our cohort, which restricts our ability to evaluate the effects of these PRS models according to individual epilepsy types.

Second, one-third of our twin cohort did not undergo rare variant testing, therefore, some concordant or discordant pairs may harbour unidentified germline pathogenic variants.

## Conclusion

Our findings indicate that the concordance for epilepsy in MZ twins is driven in part by common genetic variants, providing further evidence for the role of these variants in the liability for epilepsy. Furthermore, our data align with the liability threshold model of epilepsy, which clinicians often use in a qualitative way to guide patient counselling. This also highlights the potential utility of epilepsy PRS as a predictive marker for epilepsy risk in the general population.

## Supplementary Material

awaf362_Supplementary_Data

## Data Availability

The data that support the findings of this study are available from the corresponding author, upon reasonable request. QSKIN data will only be accessible from the QSKIN investigators through a separate application upon receiving appropriate institutional approvals.
