# Supplementary material for: Epilepsy concordance in monozygotic twins: the role of common genetic variants
Source: Brain. 2025 Sep 25;149(1):71–6. doi: 10.1093/brain/awaf362 (PMC12782162; doi:10.1093/brain/awaf362)
Supplement: awaf362_Supplementary_Data [file awaf362_supplementary_data.pdf]

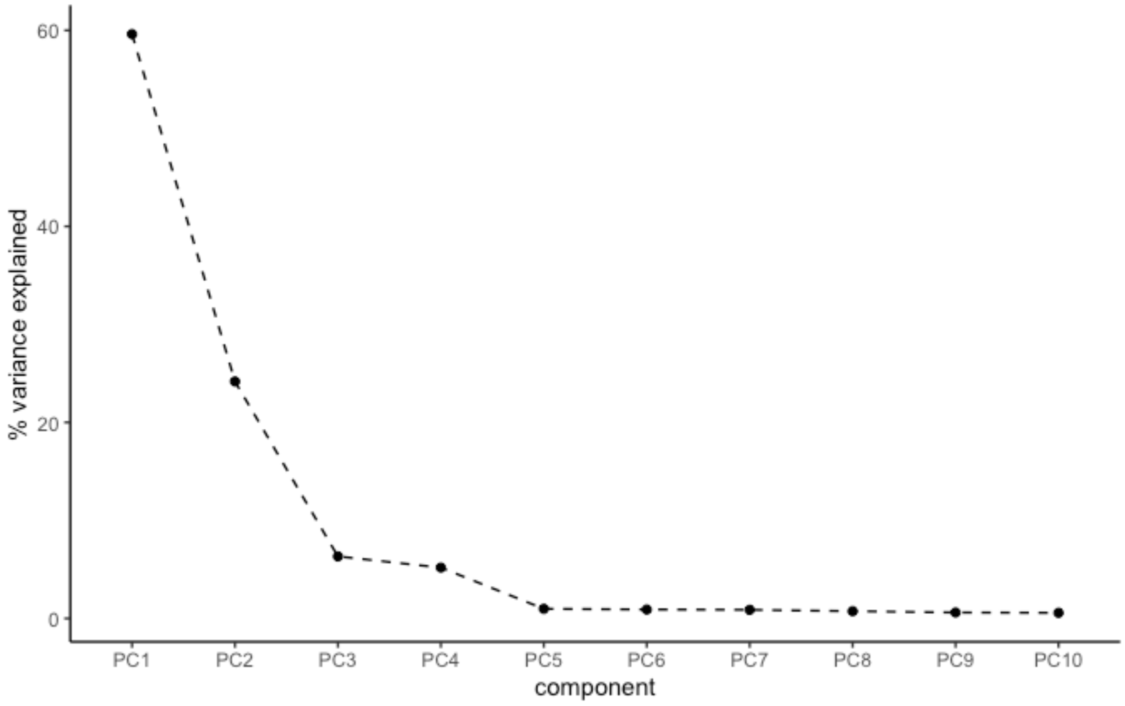

Supplementary Figure 1. Scree plot for principal components of ancestry one to ten.

Abbreviations: PC, principal component.

Supplementary Table 1. Sensitivity analysis comparing mean epilepsy PRS between concordant and discordant MZ pairs, with epilepsy type included as an additional covariate in the logistic regression model

PRS Model	Number of concordant pairs	Number of discordant pairs	**P*-value
All Epilepsy	49	53	0.03
GGE	49	53	0.05
FE	49	53	0.26

*A *p*-value of < 0.05 was considered statistically significant.

Abbreviation: FE, focal epilepsy; GGE, genetic generalized epilepsy; PRS, polygenic risk score.

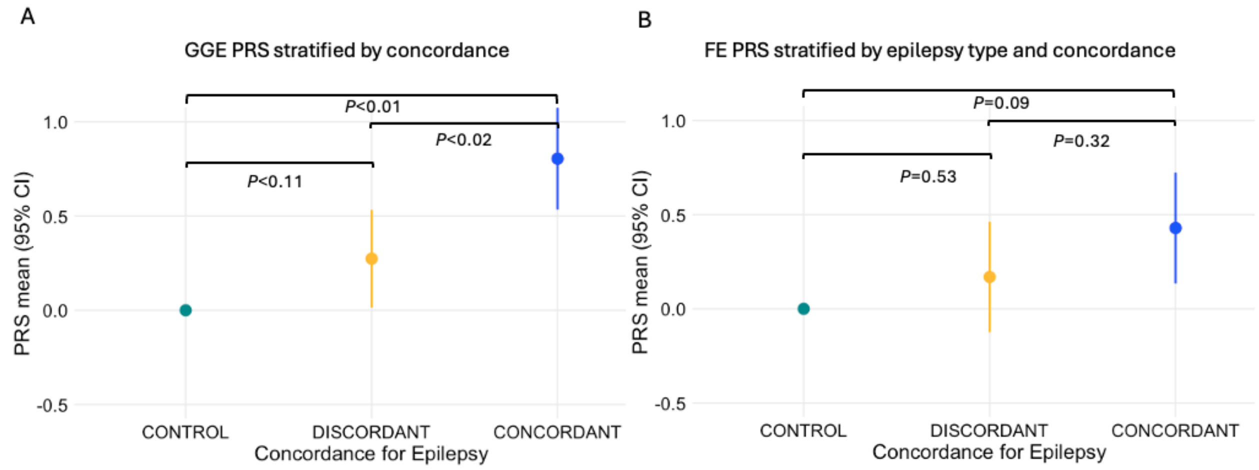

Supplementary Figure 2: A) Mean epilepsy polygenic risk scores (PRSs) with 95% confidence intervals using the 2023 Genetic Generalized Epilepsy (GGE) PRS model (for the entire cohort), stratified by concordance for epilepsy. B) Mean epilepsy polygenic risk scores (PRSs) with 95% confidence intervals using the 2023 Focal Epilepsy (FE) PRS model (for the entire cohort), stratified by concordance for epilepsy.

Mean GGE PRS in concordant twin pairs (in blue) was 0.80 (n=49), 0.27 (n=53) in discordant twin pairs (in yellow). The PRSs were normalized to a mean of 0 and a standard deviation of 1 in the control group (mean GGE PRS 0, n=14632).

Mean FE PRS in concordant twin pairs (in blue) was 0.42 (n=49), 0.17 (n=53) in discordant twin pairs (in yellow). The PRSs were normalized to a mean of 0 and a standard deviation of 1 in the control group (mean FE PRS 0, n=14632).

A p-value of <0.05 was considered statistically significant.

Abbreviations: CI, confidence interval; FE, focal epilepsy; GGE, genetic generalized epilepsy; PRS, polygenic risk score.

Supplementary Table 2. Sensitivity analysis comparing the odds ratios of MZ twin pairs concordant for epilepsy with each one-standard deviation increase in epilepsy PRS, GGE PRS or FE PRS.

PRS Model	Odds ratio	95% CI	*P-value (Wald test)
All Epilepsy	1.13	1.02-1.25	0.03
GGE	1.15	1.03-1.28	0.01
FE	1.05	0.95-1.17	0.36

*A p-value of < 0.05 was considered statistically significant.
Abbreviation: CI, confidence interval; FE, focal epilepsy; GGE, genetic generalized epilepsy; PRS, polygenic risk score.

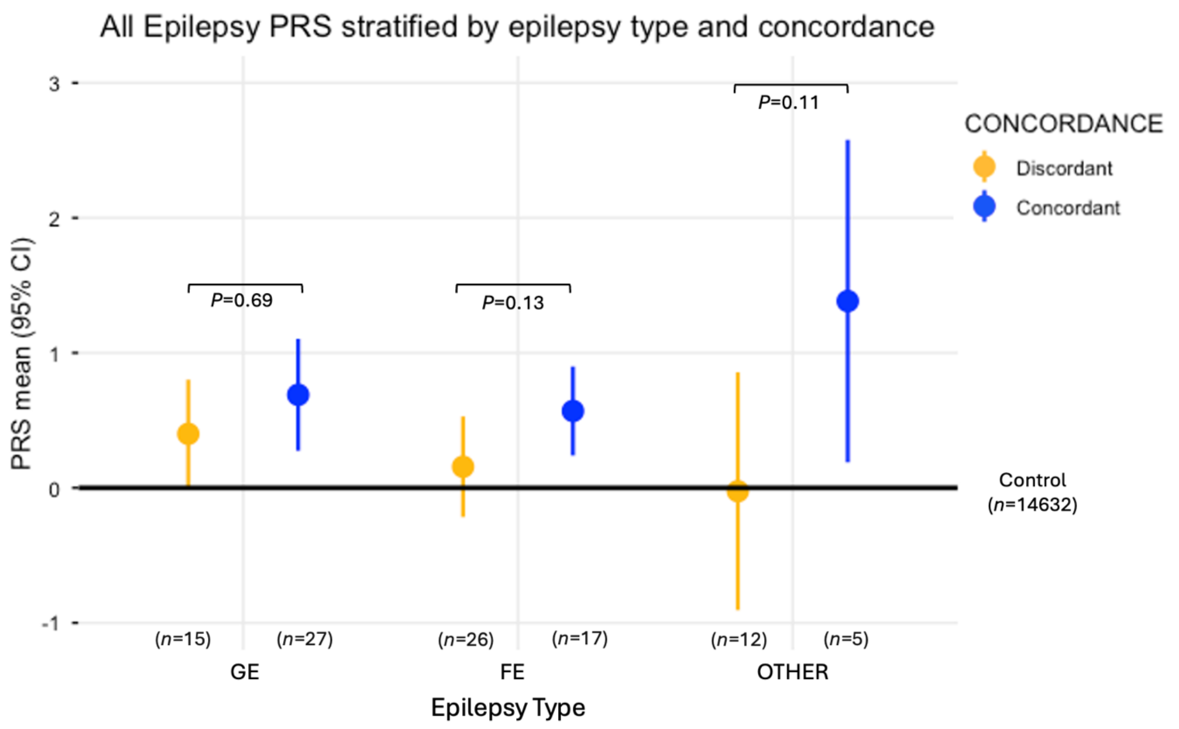

Supplementary Figure 3. Mean epilepsy polygenic risk scores (PRSs) with 95% confidence intervals using the 2023 all epilepsy PRS model, stratified by epilepsy type and concordance for epilepsy in MZ pairs.

The PRSs were standardized to a normal distribution with a mean of 0 and a standard deviation of 1 in the control group (bold horizontal line at y=0).

Comparison of mean epilepsy PRS between concordant twin pairs (in blue) and discordant twin pairs (in yellow) did not reveal significant differences for any of the epilepsy types.

A *p*-value of <0.05 was considered statistically significant.

Abbreviations: CI, confidence interval; FE; focal epilepsy; GE, generalized epilepsy; PRS, polygenic risk score.

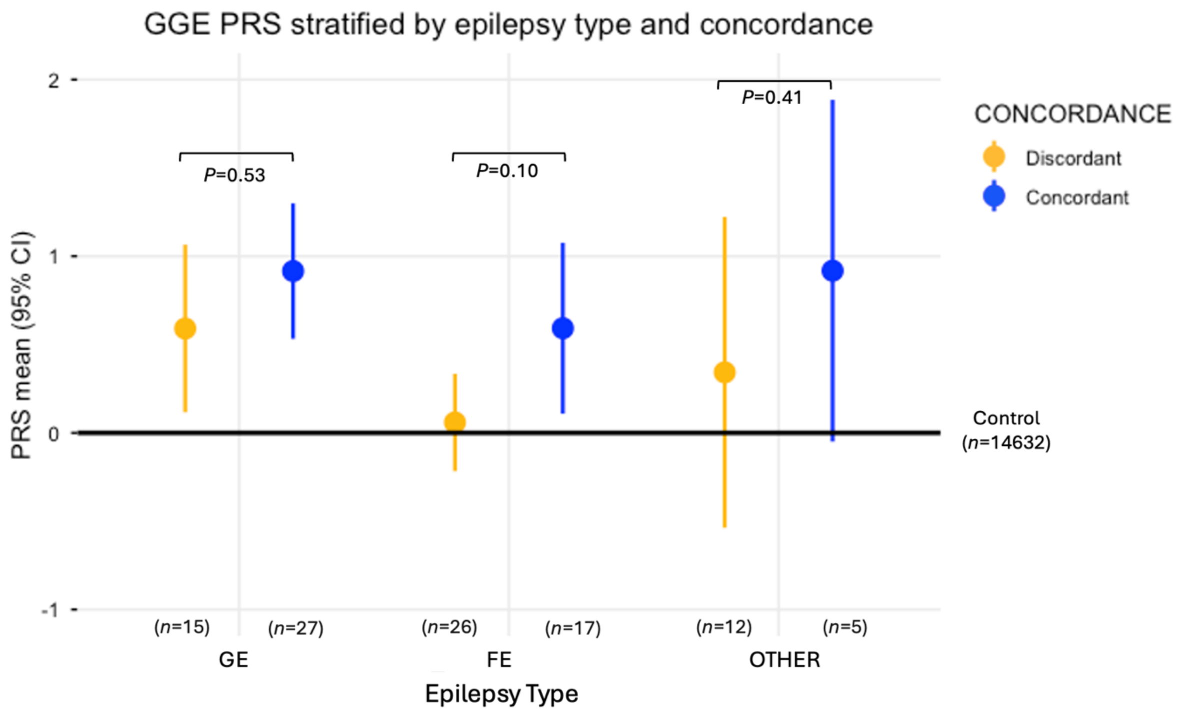

Supplementary Figure 4: Mean epilepsy polygenic risk scores (PRSs) with 95% confidence intervals using the 2023 Genetic Generalized Epilepsy (GGE) PRS model, stratified by epilepsy type and concordance for epilepsy.

The comparison of mean GGE-PRS between concordant twin pairs (in blue) and discordant twin pairs (in yellow) across all epilepsy types showed no statistically significant difference.

A *p*-value of <0.05 was considered statistically significant.

Abbreviations: CI, confidence interval; FE, focal epilepsy; GE, generalized epilepsy; GGE, genetic generalized epilepsy; PRS, polygenic risk score.

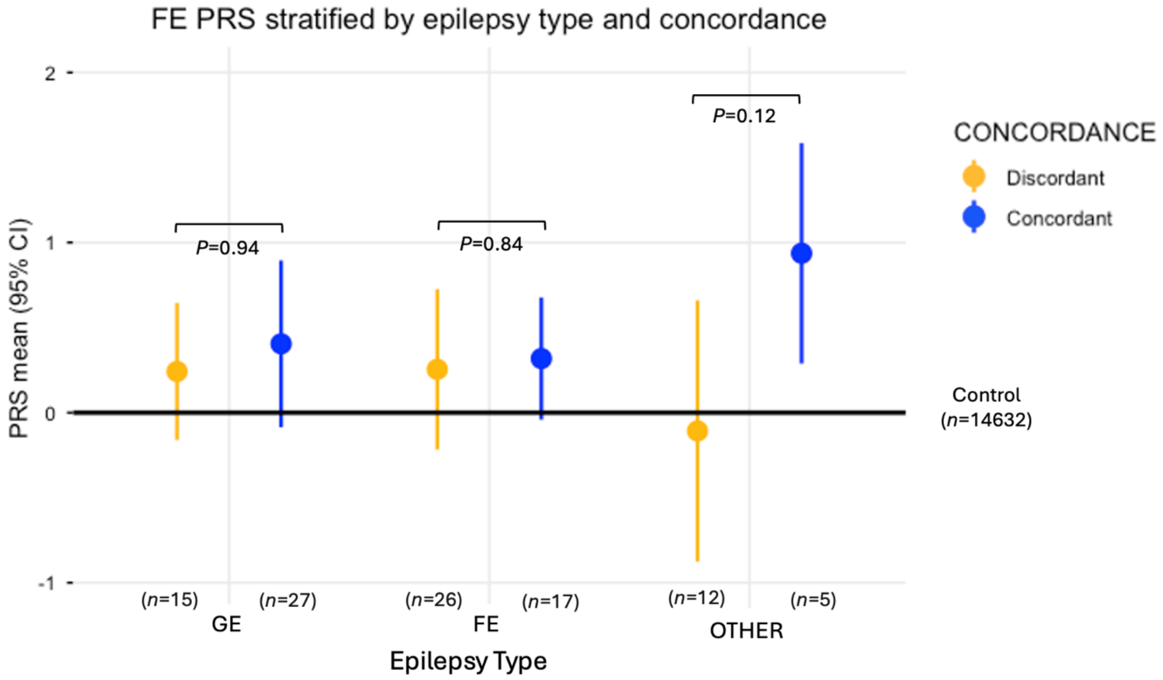

Supplementary Figure 5: Mean epilepsy polygenic risk scores (PRSs) with 95% confidence intervals using the 2023 Focal Epilepsy (FE) PRS model, stratified by epilepsy type and concordance for epilepsy.

The comparison of mean FE-PRS between concordant twin pairs (in blue) and discordant twin pairs (in yellow) across all epilepsy types revealed no statistically significant difference.

*A *p*-value of <0.05 was considered statistically significant.

Abbreviations: CI, confidence interval; FE, focal epilepsy; GE, generalized epilepsy; PRS, polygenic risk score.

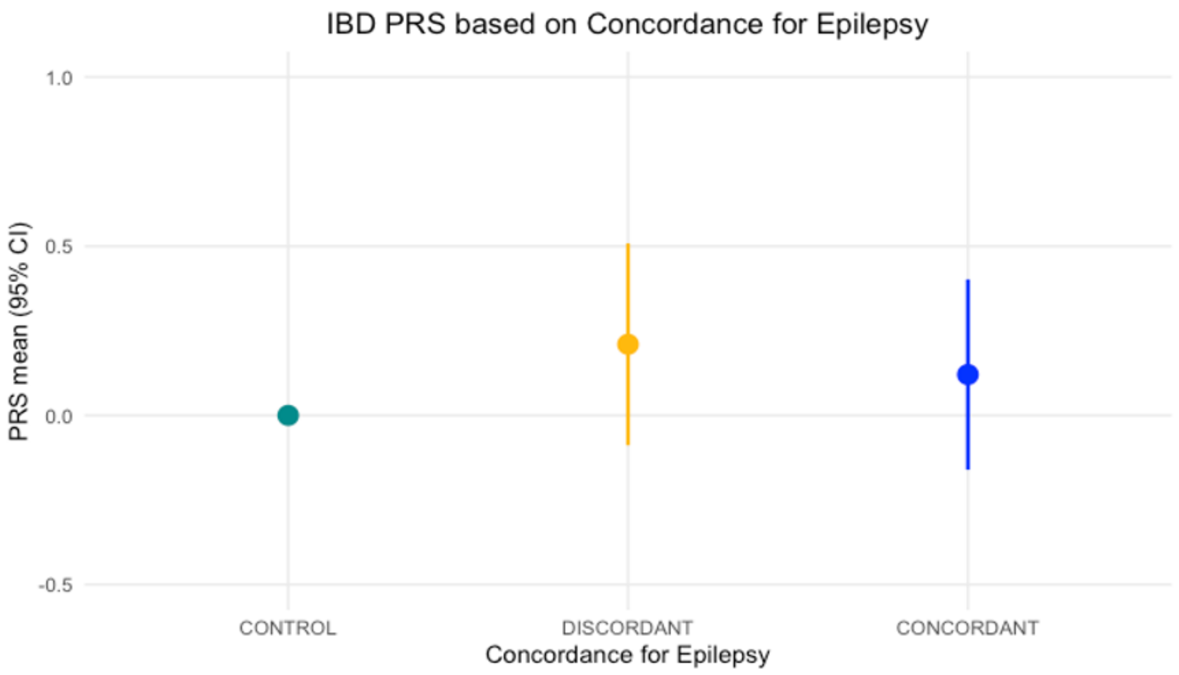

Supplementary Figure 6: A) Mean epilepsy polygenic risk scores (PRSs) with 95% confidence intervals using the 2021 Inflammatory Bowel Disease (IBD) PRS model, stratified by concordance for epilepsy.

Mean IBD PRS in concordant twin pairs (in blue) was 0.12 (*n*=49), 0.21 (*n*=53) in discordant twin pairs (in yellow). The PRSs were normalized to a mean of 0 and a standard deviation of 1 in the control group (mean IBD PRS 0, *n*=14632).

Abbreviations: CI, confidence interval; IBD, inflammatory bowel disease; PRS, polygenic risk score.
